# Supplementary material for: Neutrophils and galectin-3 defend mice from lethal bacterial infection and humans from acute respiratory failure
Source: Nat Commun. 2024 Jun 3;15:4724. doi: 10.1038/s41467-024-48796-y (PMC11148175; doi:10.1038/s41467-024-48796-y)
Supplement: Supplementary file 1 — Supplementary Information [file 41467_2024_48796_MOESM1_ESM.pdf]

Supplementary information for

Neutrophils and Galectin-3 Defend Against Lethal Bacterial Infection in Mice and Acute Respiratory Failure in Humans

Sudipta Das[1], Tomasz W Kaminski[2], Brent T. Schlegel [3,9], William Bain [1,4,9], Sanmei Hu[1], Akruti Patel[1], Sagar L. Kale[1], Kong Chen[1], Janet S. Lee[5], Rama K. Mallampalli[6], Valerian E. Kagan[7], Dhivyaa Rajasundaram[3], Bryan J. McVerry[1], Prithu Sundd[2], Georgios D. Kitsios[1,*,] Anuradha Ray [1,8,*] & Prabir Ray [1,8,10,*]

[1]Division of Pulmonary, Allergy, Critical Care, and Sleep Medicine and Acute Lung Injury Center of Excellence, Department of Medicine, University of Pittsburgh School of Medicine, Pittsburgh, PA 15213, USA
[2]VERSITI Blood Research Institute and Medical College of Wisconsin, Milwaukee, WI 53233, USA
[3]Department of Pediatrics, Division of Health Informatics, University of Pittsburgh School of Medicine, Pittsburgh, PA 15224, USA
[4]Veteran's Affairs Pittsburgh Healthcare System, Pittsburgh, PA 15240, USA
[5] Division of Pulmonary and Critical Care Medicine, Department of Medicine, Washington University School of Medicine, St. Louis, MO 63110, USA
[6]Department of Medicine, The Ohio State University (OSU), Columbus, OH 43210, USA
[7]Department of Environmental and Occupational Health, University of Pittsburgh, Pittsburgh, PA 15261, USA
[8]Department of Immunology, University of Pittsburgh School of Medicine, Pittsburgh, PA 15213, USA
[9]These authors contributed equally
[10]Lead Contact

*Correspondence: rayp@pitt.edu; raya@pitt.edu; kitsiosg@upmc.edu

This pdf file includes Supplementary Figures 1-10 and Supplementary Tables 1 and 2.

Supplementary Information List:

Supplementary Figures:

Supplementary Fig. 1. Representative lung histology of mice in different groups as shown and gating strategy to identify different innate immune cells.

Supplementary Fig. 2. Expression levels of marker genes across the identified cell types.

Supplementary Fig. 3. Immune cell clusters in each biological replicate in LPS+PA14 and PA14 mice.

Supplementary Fig. 4. Volcano plots showing the top differentially expressed genes in the different clusters (IM1, IM2, IM3, Ly6C+Lyve1lo_Mo, N3, and N4) analyzed by GSEA.

Supplementary Fig. 5. Differential enrichment of pathways based on all ontologies (biological processes, cellular components, and molecular functions) in IMs and neutrophil populations associated with response to pathogen infection in LPS+PA14 vs PA14 mice based on the GSEA data.

Supplementary Fig. 6. Pre-treatment with LPS elicits a similar outcome of infection by PA-GFP and PA14.

Supplementary Fig. 7. Parameters for identification of patients with hypo- vs hyperinflammatory status.

Supplementary Fig. 8. Clinical microbiologic culture results of lower respiratory tract specimens and associations with galectin-3 levels in research endotracheal aspirate samples.

Supplementary Fig. 9. Steroid treatment did not impact the observed associations between lower respiratory tract levels of galectin-3 and mortality by 30-days.

Supplementary Fig. 10. Adoptive transfer of CTB-labeled BAL neutrophils from WT LPS+PA14 mice into galectin-3 KO LPS+PA14 mice and gene expression in lungs of LPS+PA14 WT and KO mice.

Supplementary Tables:

Supplementary Table 1. Cell counts for different clusters.

Supplementary Table 2. Clinical characteristics of patients with acute respiratory failure, stratified by diagnosis of ARDS vs. at-risk for ARDS.

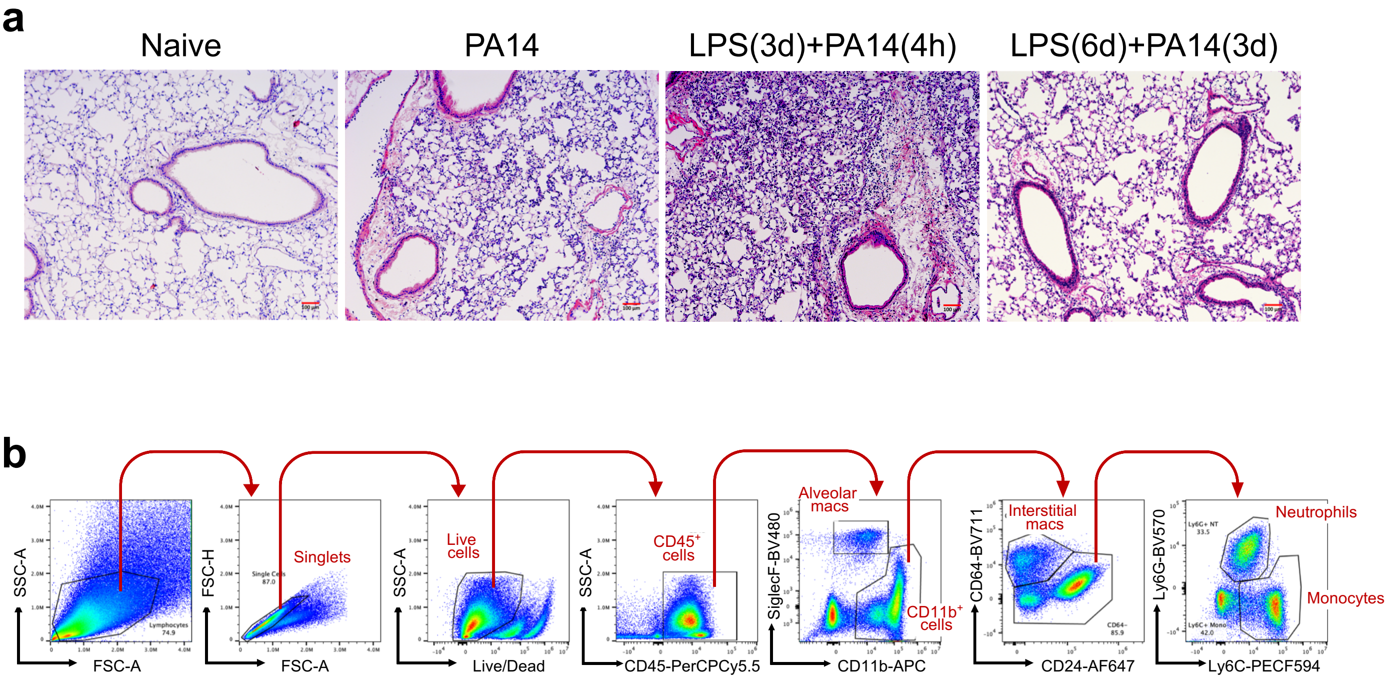

Supplementary Fig. 1.

a LPS mediated inflammation resolves over time.

Histological assessment of PA14 infected mouse lung sections by H & E staining showing LPS induced severe inflammatory response in infected mice at 4h p.i., while resolution of inflammation at 3 days p.i. Original magnification is 10X. Scale bar: 100 µm. n=3 mice per group. Data representative of 2 independent experiments.

b Gating strategy to identify different innate immune cells. Multiparameter flow cytometry density plots showing the gating strategy for different innate immune cell populations in the lungs of mice with or without PA14 infection or LPS treatment. Representative density plots taken from multiple groups to show all different cell populations.

a

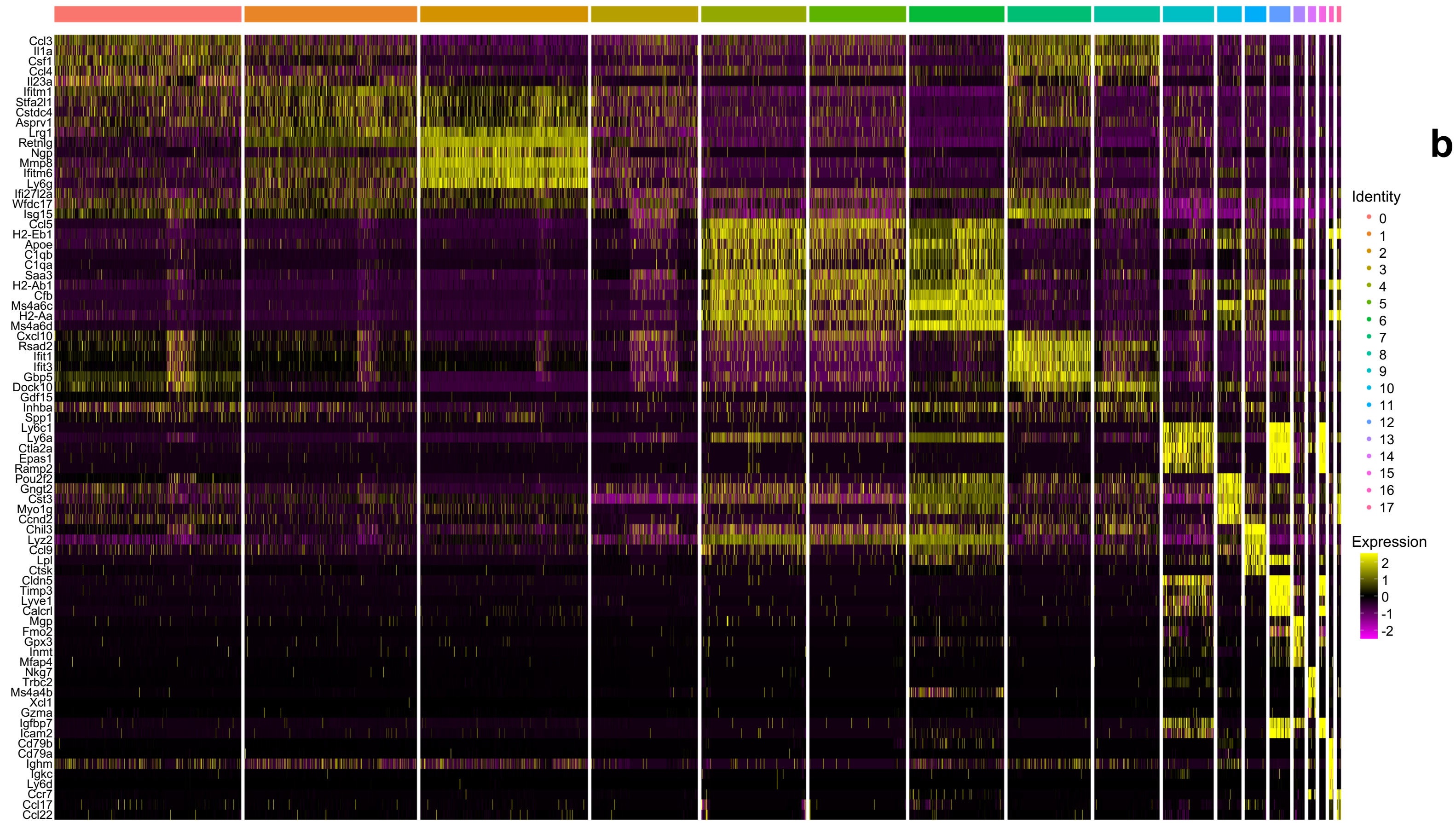

b

Annotation	Cluster
N4	0
N4	1
N1	2
N2	3
IM2	4
IM2	5
IM3	6
N3	7
N3	8
Ly6C+Lyve1lo_Mo	9
FABP_M	10
AM	11
IM1	12
Fibroblast	13
NK	14
Ly6C+Lyve1lo_Mo	15
B-Cell	16
DC	17

c

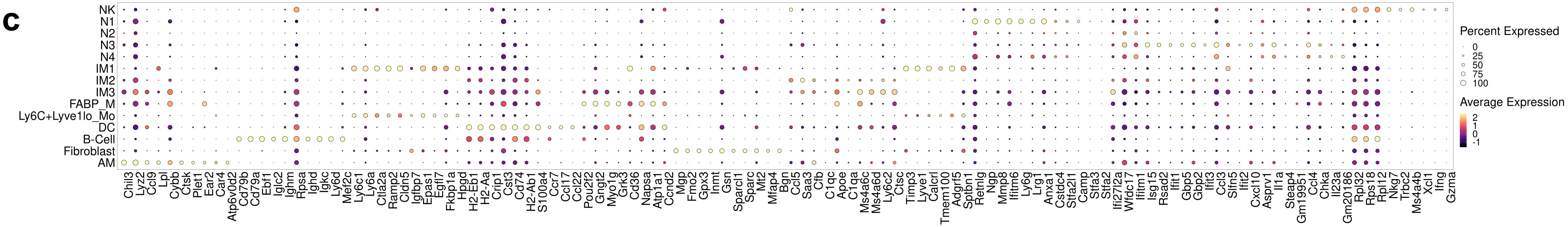

Supplementary Fig. 2. Expression levels of marker genes across the identified cell types. a Heat map depicting the marker genes corresponding to 18 clusters. Similar clusters were merged resulting in 14 clusters (shown in Fig. 3) with distinct marker gene expression. **b** Table showing corresponding annotations for each cluster. **c** Expression levels of marker genes in the 14 clusters. The size of each dot indicates the proportion of cells of each type expressing the specific gene and is colored to indicate the average expression of the gene across all cells as shown in the legend.

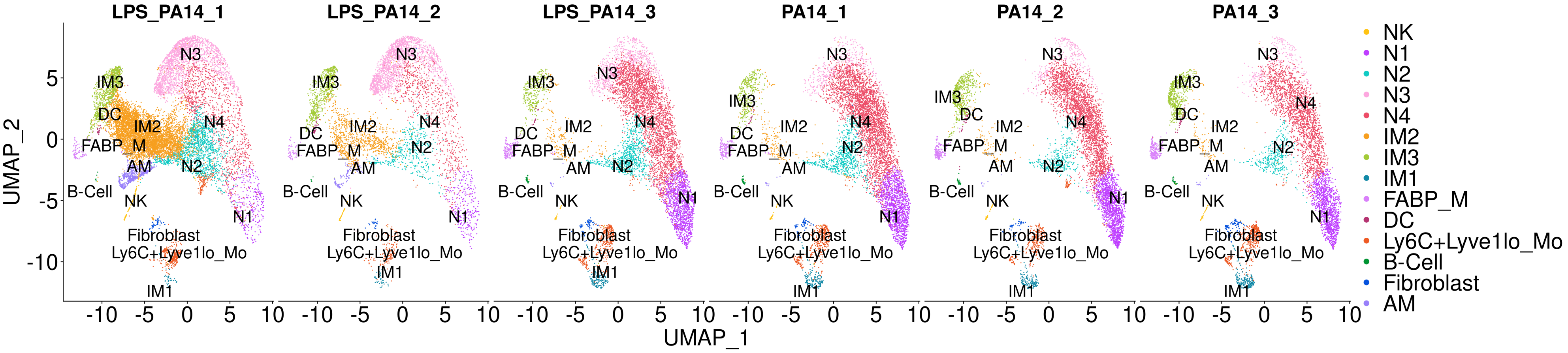

Supplementary Fig. 3. Identification of immune cell populations in lungs of individual replicates of LPS+PA14 and PA14 mice by scRNA-seq . The split 'dimplot' shown to represent consistency of the UMAP based on replicates.

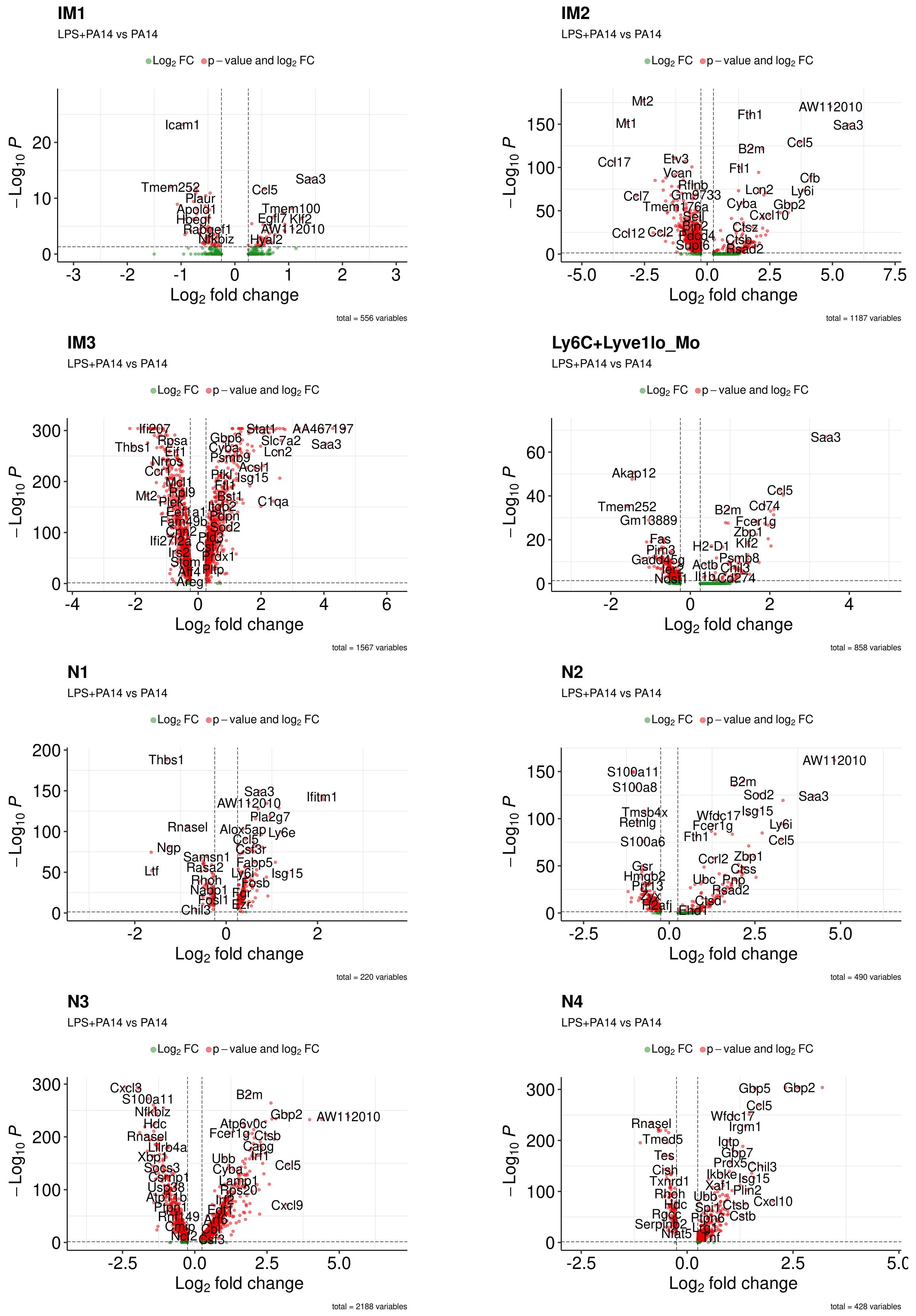

Supplementary Fig. 4. Volcano plots showing the top differentially expressed genes in the different clusters (IM1, IM2, IM3, Ly6C+Lyve1lo_Mo, N3, and N4) analyzed by GSEA. The significant DEGs with both satisfying conditions of p-values < 0.05 and logFC ≥ 0.25 and logFC ≤ 0.25 are indicated by red dots with gene names. Green dots indicate the remaining genes present that were not significantly changed. The genes that are upregulated are on the right panel, and downregulated ones are on the left panel of the plots.

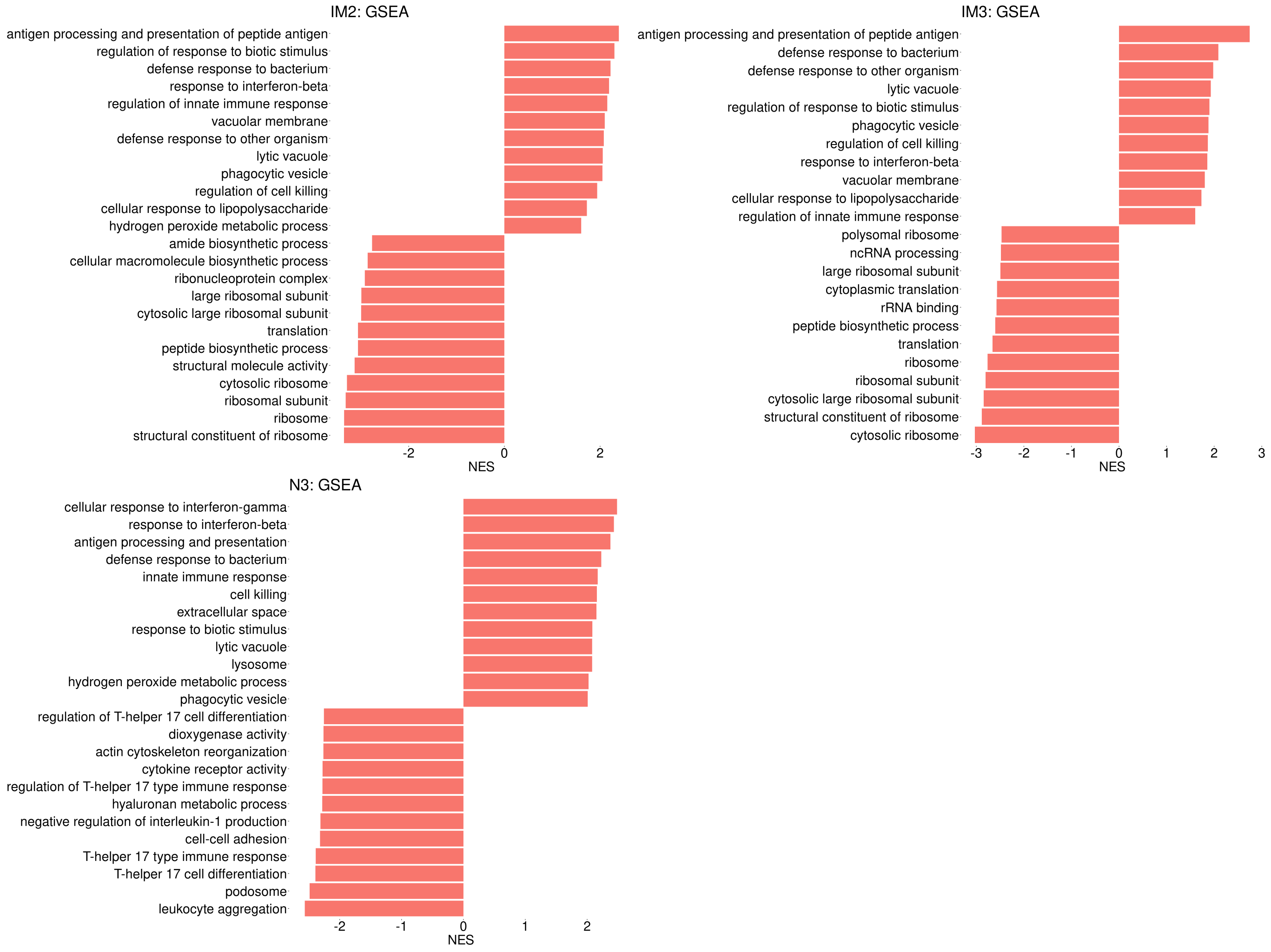

Supplementary Fig. 5. Differential enrichment of pathways based on all ontologies (biological processes, cellular components, and molecular functions) in IMs and neutrophil populations associated with response to pathogen infection in LPS+PA14 vs PA14 mice based on the GSEA data. The bars to the right and left, represent, respectively, a positive and negative enrichment in the associated pathway, and the x-axis shows the NES of the GSEA analysis, and the y-axis indicates the enriched pathways.

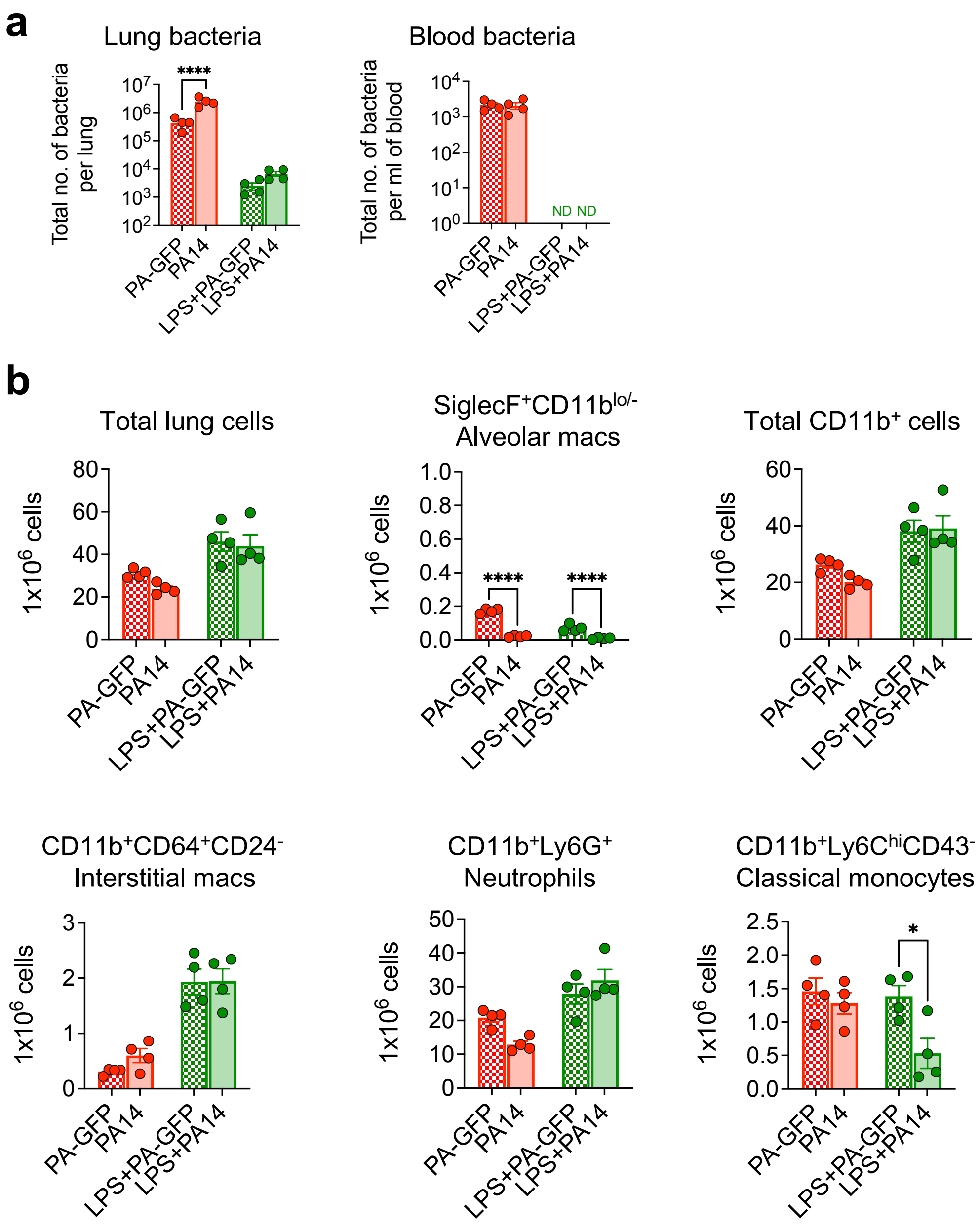

Supplementary Fig. 6. Pre-treatment with LPS elicits a similar outcome of infection by PA-GFP and PA14. a Similar bacterial load in the lung and peripheral blood in response to bacterial infection by either PA-GFP or PA14 (PA-GFP and PA14) and lower but similar response with prior LPS pre-exposure when infected by either PA strain (LPS+PA-GFP or LPS+PA14). Data were log transformed using log base 10 to adjust for differences in standard deviation prior to analysis. **b** Flow cytometry analysis showing cell counts in the lungs of four groups of mice infected by PA14 or PA-GFP without or with LPS pre-exposure. n=2 mice per group. **(a and b)** Data pooled from 2 independent experiments and analyzed using ordinary one-way ANOVA with the Sidak's test. All data are presented as mean ± s.e.m. *P < 0.05, ****P < 0.0001

a Parsimonious logistic regression model for phenotypic assignments

Intercept	IL-6	Serum bicarbonate	sTNFR1
-11.9593	1.0138	-0.2436	1.1903

b Biomarker variable comparisons by phenotypes

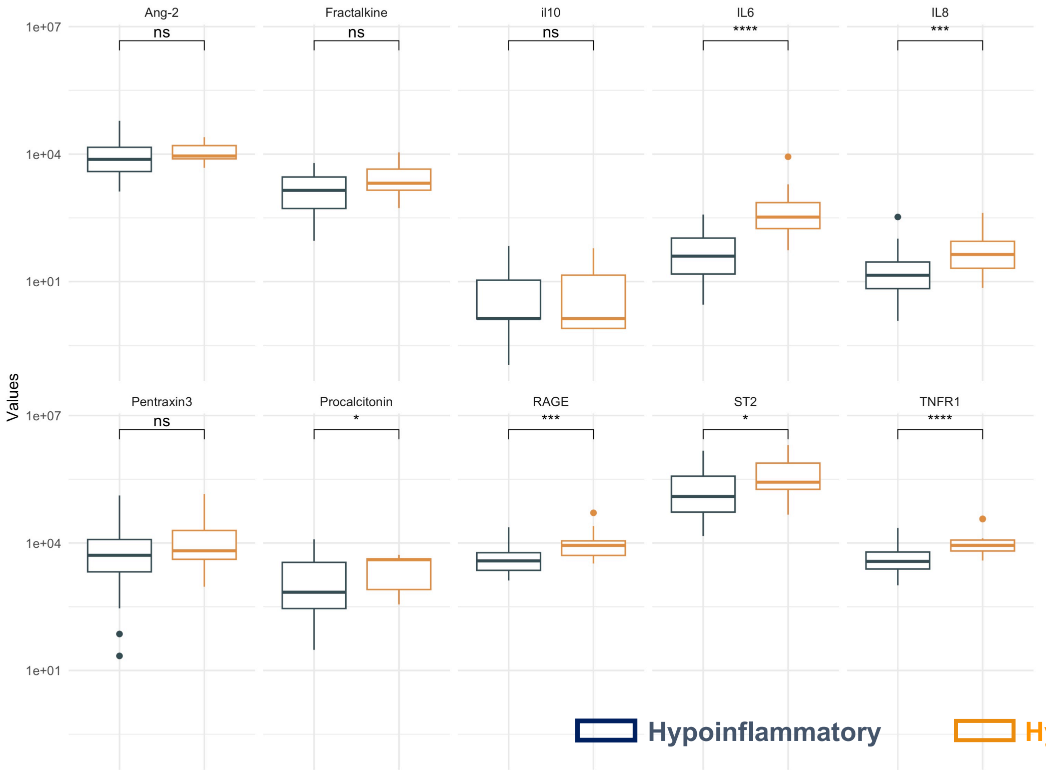

c Clinical variable comparisons by phenotypes

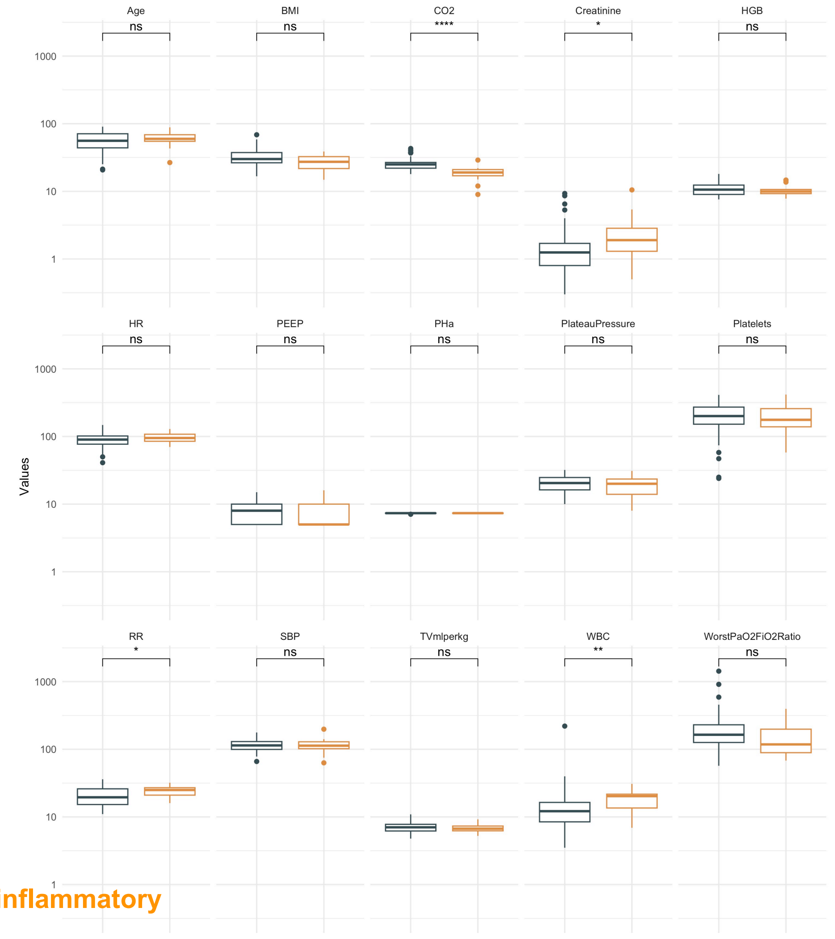

Supplementary Fig. 7. Parameters for identification of patients with hypo vs. hyperinflammatory status. a Phenotype classification methodology. As shown in Table, the model utilized plasma levels of two biomarkers (IL-6 and sTNFR1) and clinically available serum bicarbonate levels. Predicted probabilities from this parsimonious logistic regression model was used to classify patients to a hypo vs. hyperinflammatory phenotype using the Youden index cutoff of 0.274. **b** Plasma biomarker comparisons between phenotypes (black: hypoinflammatory; orange: hyperinflammatory). Comparisons were performed with Wilcoxon tests and Y-axes in each graph are displayed with $\log_{10}$ transformations. Apart from the two defining variables (IL-6 and sTNFR1), hyperinflammatory patients had statistically significant higher levels of IL-8, procalcitonin, RAGE and ST2 compared to hypoinflammatory patients (all $p<0.05$). **c** Clinical variable comparisons between phenotypes. Comparisons were performed with Wilcoxon tests and Y-axes in each graph are displayed with $\log_{10}$ transformations. Apart from the defining variable CO_2 (serum bicarbonate), hyperinflammatory patients had significantly higher creatinine and white blood cell count levels, as well as a higher total respiratory rate at baseline. Statistical significance annotations: *:<0.05; **: <0.01; ***: <0.001; ****: <0.0001. Abbreviations: Ang-2: Angiopoietin-2; IL: interleukin; RAGE: receptor of advanced glycation end-products; ST2: suppressor of tumorigenicity-2; sTNFR1: soluble tumor necrosis factor receptor 1; BMI: body mass index; HGB: hemoglobin; HR: hear rate; PEEP: positive end expiratory pressure; PHa: PH arterial blood gas; RR: Respiratory Rat; SBP: Systolic blood pressure; TVmlperkg: Tidal volume per kilogram of ideal body weight; WBC: White blood cell count; WorstPaO2FiO2Ratio: lowest ratio of partial pressure of oxygen in arterial blood gas over inspired concentration of oxygen.

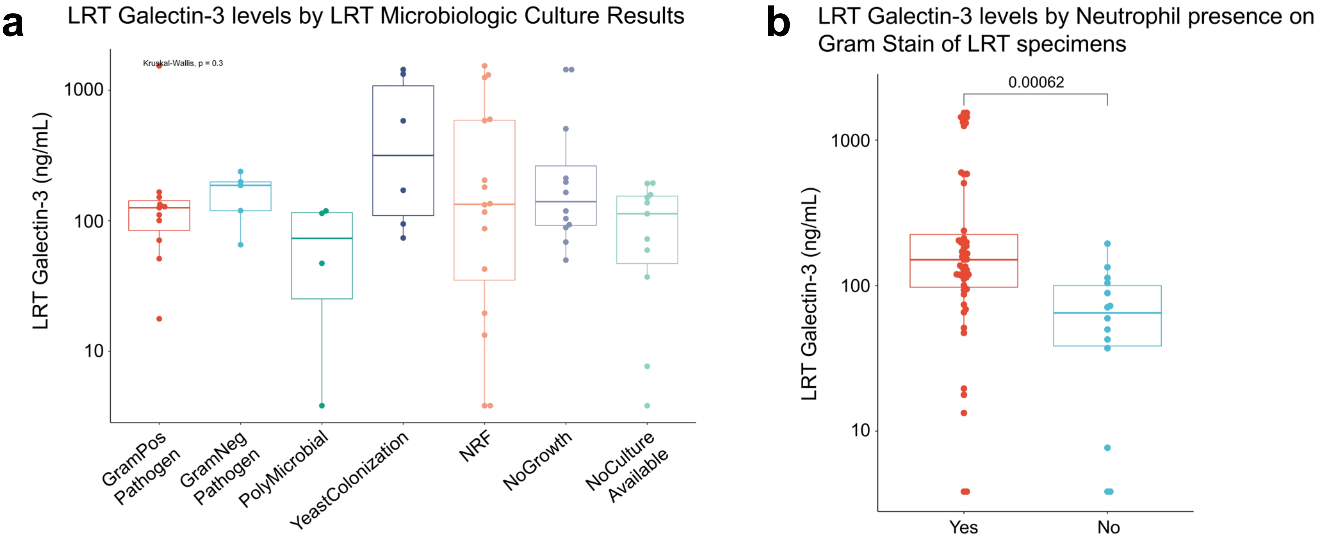

Supplementary Fig. 8. Clinical microbiologic culture results of lower respiratory tract specimens and associations with galectin-3 levels in research endotracheal aspirate samples. a No significant differences of LRT galectin-3 levels by isolated organisms in clinical microbiology analyses. Gram-positive pathogens included *S.aureus* (n=8) and *Streptococcus spp* (n=2), whereas gram-negative pathogens included *P.aeruginosa* (n=1) and *Enterobacteriaceae* (n=4). **b** Presence of neutrophils on gram stain analysis of clinical LRT biospecimens was strongly associated with galectin-3 levels in synchronous (within 48hrs) research ETA biospecimens.

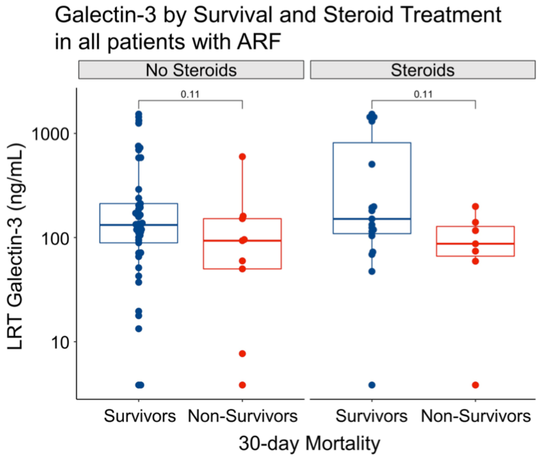

Supplementary Fig. 9. Steroid treatment did not impact the observed associations between lower respiratory tract levels of galectin-3 and mortality by 30-days. Upon stratification by use of steroids at baseline, no significant differences in the relationship of galectin-3 levels with 30-day mortality was found.

a

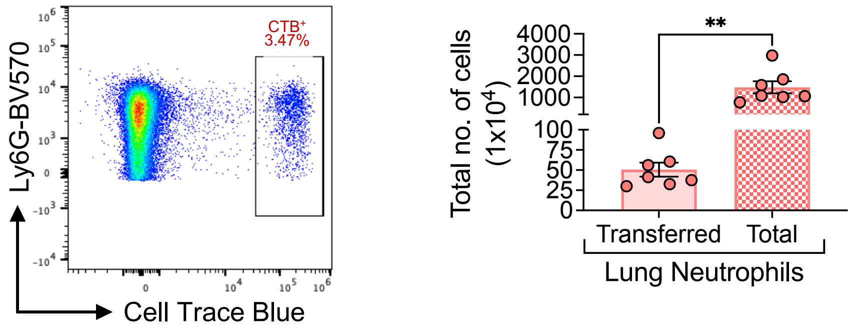

b

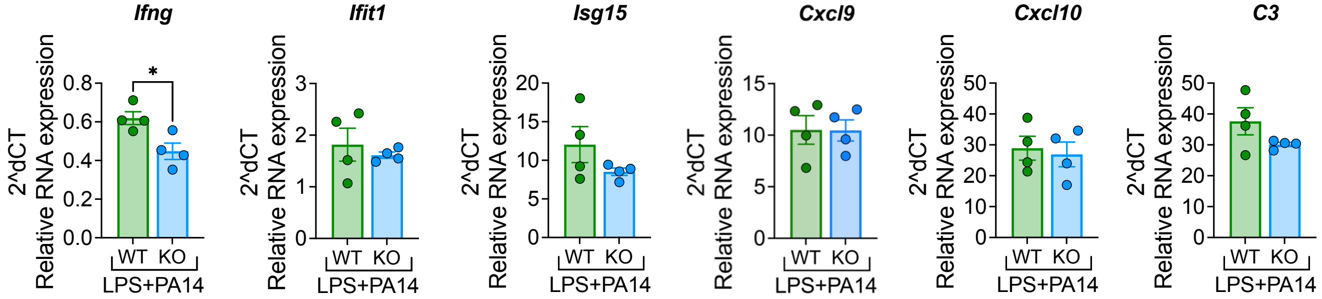

Supplementary Fig. 10.

a Efficient adoptive transfer and survival of WT neutrophils in Gal3-KO lungs assessed by flow cytometry. Representative flow plot for identification of adoptively transferred cell trace blue (CTB) labeled WT neutrophils in Gal3-KO lungs. Flow cytometry analysis showing number of transferred neutrophils vs total neutrophils in lungs of LPS pre-exposed Gal3-KO infected mice. n=7 mice total per group. Data pooled from 2 independent experiments and analyzed using two-tailed unpaired t-test with Welch's correction.

b Lack of Galectin-3 downregulates *Ifng* expression without altering the expression of other relevant genes. RT-qPCR analysis of gene expression of *Ifng*, *Ifit1*, *Isg15, Cxcl9, Cxcl10 and C3* complement in the lung tissue of the two groups of mice. n=4 mice per group. Data representative of 3 independent experiments and analyzed using two-tailed unpaired t-test with Welch's correction.

Supplementary Table 1. Cell counts for different clusters.

Cell type	Condition	Count
NK	LPS+ PA14	186
NK	PA14	114
N1	LPS+ PA14	1975
N1	PA14	4394
N2	LPS+ PA14	2592
N2	PA14	1472
N3	LPS+ PA14	4791
N3	PA14	869
N4	LPS+ PA14	5089
N4	PA14	8555
IM1	LPS+ PA14	231
IM1	PA14	566
IM2	LPS+ PA14	7244
IM2	PA14	412
IM3	LPS+ PA14	1982
IM3	PA14	1631
FABP.M	LPS+ PA14	337
FABP.M	PA14	594
Ly6C+Lyve1lo.Mo	LPS+ PA14	1007
Ly6C+Lyve1lo.Mo	PA14	1191
DC	LPS+ PA14	87
DC	PA14	81
B-Cell	LPS+ PA14	49
B-Cell	PA14	126
Fibroblast	LPS+ PA14	168
Fibroblast	PA14	269
AM	LPS+ PA14	785
AM	PA14	29

Supplementary Table 2. Clinical characteristics of patients with acute respiratory failure, stratified by diagnosis of ARDS vs. at-risk for ARDS.

Abbreviations: LIPS: lung injury prediction score; BMI: body mass index; WBC: white blood cell count; SOFA: sequential organ failure assessment score; VFDs: ventilator-free days; ETA endotracheal aspirate.

P values for comparisons of continuous variables are from Wilcoxon test, whereas p-values for categorical variables are from Fisher's exact tests. All tests were two-sided.

	**ARDS**	**At-risk for ARDS**	**p**
**n**	28	53	
Age, years, median (IQR)	52.5 [40.9, 59.8]	59.1 [51.9, 72.2]	0.02
Men, n (%)	14 (50.0)	36 (67.9)	0.18
Diabetes, n (%)	11 (39.3)	24 (45.3)	0.78
Immunosuppressed, n (%)	9 (32.1)	5 (9.4)	0.02
COPD, n (%)	1 (3.6)	11 (20.8)	0.08
Pneumonia, n (%)	16 (59.3)	36 (67.9)	0.6
Aspiration, n (%)	3 (11.1)	16 (30.2)	0.11
Extra-pulmonary sepsis, n (%)	3 (11.1)	16 (30.2)	0.11
LIPS score, median (IQR)	6.0 [5.2, 7.0]	6.0 [5.0, 7.2]	0.93
BMI, median (IQR)	29.6 [27.1, 35.0]	28.9 [25.1, 35.9]	0.51
WBC, $x10^9/L$, median (IQR)	12.3 [8.9, 18.1]	13.4 [9.1, 18.6]	0.72
Creatinine, mg/dL, median (IQR)	1.1 [0.8, 1.6]	1.5 [1.0, 2.3]	0.14
Worst PaO2/FiO2, median (IQR)	150.0 [84.0, 190.0]	164.0 [134.5, 247.0]	0.04
SOFA score, median (IQR)	6.0 [5.0, 8.0]	7.0 [5.0, 8.0]	0.61
VFDs, median (IQR)	19.5 [11.0, 25.0]	22.0 [15.5, 25.0]	0.52
30-day mortality, n (%)	6 (21.4)	10 (18.9)	1
Steroid treatment at baseline, n (%)	9 (52.9)	17 (65.4)	0.62
Prednisone daily dose equivalent (mg) at baseline, median (IQR)	8.0 [0.0, 60.0]	35.0 [0.0, 71.0]	0.47
ETA Galectin-3, ng/ml, median (IQR)	146.7 [83.4, 613.1]	119.1 [71.3, 193.1]	0.25
ETA Neutrophil Elastase, ug/ml, median (IQR)	674.2 [100.8, 1564.8]	124.6 [31.2, 495.6]	0.03
