# Supplementary material for: Neutrophils and galectin-3 defend mice from lethal bacterial infection and humans from acute respiratory failure
Source: Nat Commun. 2024 Jun 3;15:4724. doi: 10.1038/s41467-024-48796-y (PMC11148175; doi:10.1038/s41467-024-48796-y)
Supplement: Supplementary file 3 — Description of Additional Supplementary Files [file 41467_2024_48796_MOESM3_ESM.pdf]

Description of Additional Supplementary Files

Supplementary Data Legends

Supplementary Data 1 : GSEA data for each cluster LPS+PA14 vs PA14. The data were generated using clusterProfiler and can be interpreted using the normalized enrichment scores (NES), and BH adjusted p-values p-values ($p < 0.05$) The list of enriched core genes of each pathway is also included.

Supplementary Movie Legends

Supplementary Movie 1 : (Vehicle+PA). Quantitative fluorescence intravital lung microscopy (qFILM) was used to assess phagocytosis of P. aeruginosa (PA) by neutrophils in the lungs of live mice that received vehicle (PBS) and infected. Video recordings were made 3 h post infection with eGFP-labeled PA. The microcirculation (pseudo-colored purple) and neutrophils (pseudo-colored red) were labeled in vivo by IV administration of RedTexas dextran and Pacific Blue-antiLy6G Ab, respectively. Please refer to detailed methods in main text. Representative qFILM video of lung microcirculation shows erythrocytes (dark, unstained cells) and neutrophils (red) rapidly transiting through the pulmonary microcirculation. Neutrophils (red) attached to vessel walls belong to the marginated pool of neutrophils. The colocalization of neutrophils (red) and eGFP-PA (green) is rare, despite the presence of eGFP-PA in the lung microcirculation. Scale bar 20 µm. Video shown in an original acquisition rate.

Supplementary Movie 2 : (LPS+PA). Quantitative fluorescence intravital lung microscopy (qFILM) was used to assess phagocytosis of P. aeruginosa (PA) by neutrophils in the lungs of live, LPS-pretreated mice 3 h post infection with eGFP-labeled PA. The microcirculation (pseudo-colored purple) and neutrophils (pseudo-colored red) were labeled in vivo by IV administration of RedTexas dextran and Pacific Blue-antiLy6G Ab, respectively. Representative qFILM video of lung microcirculation shows the red neutrophil crawling intravascularly over 1 minute to initiate the phagocytosis of eGFP-PA (green color). Within the next 30 seconds the colocalization of neutrophil (red) and eGFP-PA (green) is observed as a blended yellow color. Scale bar 10 µm. Video shown in 25x original acquisition rate to capture phagocytic events.
