# Supplementary material for: Neutrophils and galectin-3 defend mice from lethal bacterial infection and humans from acute respiratory failure
Source: Nat Commun. 2024 Jun 3;15:4724. doi: 10.1038/s41467-024-48796-y (PMC11148175; doi:10.1038/s41467-024-48796-y)
Supplement: Supplementary file 7 — Reporting Summary [file 41467_2024_48796_MOESM7_ESM.pdf]

nature portfolio

Prabir Ray (rayp@pitt.edu)
Anuradha Ray (raya@pitt.edu)
Georgios Kitsios (kitsiosg@upmc.edu)
Corresponding author(s):

Last updated by author(s): 2024-04-15

Reporting Summary

Nature Portfolio wishes to improve the reproducibility of the work that we publish. This form provides structure for consistency and transparency in reporting. For further information on Nature Portfolio policies, see our Editorial Policies and the Editorial Policy Checklist.

Statistics

For all statistical analyses, confirm that the following items are present in the figure legend, table legend, main text, or Methods section.

n/a	Confirmed	
☐	☒	The exact sample size (n) for each experimental group/condition, given as a discrete number and unit of measurement
☒	☐	A statement on whether measurements were taken from distinct samples or whether the same sample was measured repeatedly
☐	☒	The statistical test(s) used AND whether they are one- or two-sided<br>*Only common tests should be described solely by name; describe more complex techniques in the Methods section.*
☐	☒	A description of all covariates tested
☐	☒	A description of any assumptions or corrections, such as tests of normality and adjustment for multiple comparisons
☐	☒	A full description of the statistical parameters including central tendency (e.g. means) or other basic estimates (e.g. regression coefficient) AND variation (e.g. standard deviation) or associated estimates of uncertainty (e.g. confidence intervals)
☐	☒	For null hypothesis testing, the test statistic (e.g. F, t, r) with confidence intervals, effect sizes, degrees of freedom and P value noted<br>*Give P values as exact values whenever suitable.*
☒	☐	For Bayesian analysis, information on the choice of priors and Markov chain Monte Carlo settings
☒	☐	For hierarchical and complex designs, identification of the appropriate level for tests and full reporting of outcomes
☒	☐	Estimates of effect sizes (e.g. Cohen's d, Pearson's r), indicating how they were calculated

Our web collection on statistics for biologists contains articles on many of the points above.

Software and code

Policy information about availability of computer code

Data collection	Standard data collection methods described in published literature were used in this study
Data analysis	standard analysis methods described in published literature were used.

For manuscripts utilizing custom algorithms or software that are central to the research but not yet described in published literature, software must be made available to editors and reviewers. We strongly encourage code deposition in a community repository (e.g. GitHub). See the Nature Portfolio guidelines for submitting code & software for further information.

Data

Policy information about availability of data

All manuscripts must include a data availability statement. This statement should provide the following information, where applicable:

- Accession codes, unique identifiers, or web links for publicly available datasets
- A description of any restrictions on data availability
- For clinical datasets or third party data, please ensure that the statement adheres to our policy

All scRNA-seq generated in this study are available in the GEO with accession number GSE237646.
https://www.ncbi.nlm.nih.gov/geo/query/acc.cgi?acc=GSE237646

Research involving human participants, their data, or biological material

Policy information about studies with human participants or human data. See also policy information about sex, gender (identity/presentation), and sexual orientation and race, ethnicity and racism.

Reporting on sex and gender	We recorded participant's sex as reported in the electronic medical record. We have included disaggregated data by sex in the Source Data file. We obtained informed consent by participants for sharing de-identified data. We did not perform further analyses stratified by sex due to small sample size.
Reporting on race, ethnicity, or other socially relevant groupings	Details included in manusript
Population characteristics	All information included in Supplementary Table 3.
Recruitment	Following admission to the ICU and obtaining informed consent from patients or their legally authorized representatives (IRB protocol STUDY19050099), we collected baseline research endotracheal aspirates (ETA) biospecimens within 72hrs from intubation.
Ethics oversight	University of Pittsburgh, IRB protocol STUDY19050099

Note that full information on the approval of the study protocol must also be provided in the manuscript.

Field-specific reporting

Please select the one below that is the best fit for your research. If you are not sure, read the appropriate sections before making your selection.

☒ Life sciences ☐ Behavioural & social sciences ☐ Ecological, evolutionary & environmental sciences

For a reference copy of the document with all sections, see nature.com/documents/nr-reporting-summary-flat.pdf

Life sciences study design

All studies must disclose on these points even when the disclosure is negative.

Sample size	For mouse studies, power analysis was performed based on the differential bacterial load between the LPS+PA14 and PA14 mice which yielded a sample size of 3 in each group.For the human study, a consensus committee of ≥ 3 physician-scientists certified in critical care reviewed clinical and radiographic data and performed retrospective classifications of the etiology and severity of ARF without any knowledge of experimental data or outcomes.
Data exclusions	No data excluded.
Replication	All mouse experiments, experiments were repeated at least once or twice yielding reproducible data.
Randomization	It was difficult to perform the mouse study in a randomized and blinded fashion. This is because LPS-treated or not and infected mice had to be grouped separately and monitored for survival continuously. AS mentioned above, for the human study, a consensus committee of ≥ 3 physician-scientists certified in critical care reviewed clinical and radiographic data and performed retrospective classifications of the etiology and severity of ARF without any knowledge of experimental data or outcomes.
Blinding	

Reporting for specific materials, systems and methods

We require information from authors about some types of materials, experimental systems and methods used in many studies. Here, indicate whether each material, system or method listed is relevant to your study. If you are not sure if a list item applies to your research, read the appropriate section before selecting a response.

Materials & experimental systems

n/a	Involved in the study	
☐	☒	Antibodies
☒	☐	Eukaryotic cell lines
☒	☐	Palaeontology and archaeology
☐	☒	Animals and other organisms
☐	☒	Clinical data
☒	☐	Dual use research of concern
☒	☐	Plants

Methods

n/a	Involved in the study	
☒	☐	ChIP-seq
☐	☒	Flow cytometry
☒	☐	MRI-based neuroimaging

Antibodies

Antibodies used	The fluorochrome-conjugated antibodies used were: anti-mouse CD45-PerCP/Cyanine5.5 (Dilution: 1:500) (Clone: 30-F11) (Biolegend, Cat# 103131), SiglecF-BV480 (Dilution: 1:500) (Clone: E50-2440) (BD Biosciences, Cat# 746668), CD11b-APC (Dilution: 1:500) (Clone: M1/70) (Biolegend, Cat# 101211), CD11c-BV785 (Dilution: 1:500) (Clone: N418) (Biolegend, Cat# 117335), CD64-BV711 (Dilution: 1:500) (Clone: X54-5/7.1) (Biolegend, Cat# 139311), CD24-AlexaFluor647(Dilution: 1:500) (Clone: M1/69) (Biolegend, Cat# 101818), Ly6G-BV570 (Dilution: 1:500) (Clone: 1A8) (Biolegend, Cat# 127629), Ly6C-PE-CF594 (Dilution: 1:500) (Clone: AL-21) (BD Biosciences, Cat# 562728), CD43-APC-R700 (Dilution: 1:500) (Clone: S7) (BD, Cat# 565532), CD14-BV510 (Dilution: 1:500) (Clone: Sa14-2) (Biolegend, Cat# 123323). Intracellular staining for cytokine was carried out using IFN-γ-FITC (Dilution: 1:500) (Clone: XMG1.2) (BD Biosciences, Cat# 554411) antibody in Foxp3 transcription factor staining buffer (eBioscience, Cat# 00-5523-00) according to manufacturer's protocol. To assess cell viability, FVD EFluor 780 (Dilution: 1:3000) (eBioscience, Cat# 65-0865-14); mouse Fc block (BD Biosciences, Cat# 553142).
Validation	All antibodies used were validated by the manufacturers as noted on their websites.

Animals and other research organisms

Policy information about studies involving animals; ARRIVE guidelines recommended for reporting animal research, and Sex and Gender in Research

Laboratory animals	C57BL/6J mice (Cat# 000664) and B6.Cg-Lgals3tm1Poi/J (Cat# 006338)purchased from The Jackson Laboratory were used. Mice used were 10-12 weeks old. All animals were housed in approved animal facilities (Division of Laboratory Animal Resources) of the University of Pittsburgh where temperature, humidity, lighting, and sanitation are controlled. Animals were maintained on a 12 h light and 12 h dark cycle, 55-60% humidity and 20-24C.
Wild animals	
Reporting on sex	LPS-mediated protection was observed in both sexes.
Field-collected samples	N/A
Ethics oversight	Institutional Animal Care and Use Committee (IACUC) at the University of Pittsburgh.

Note that full information on the approval of the study protocol must also be provided in the manuscript.

Clinical data

Policy information about clinical studies

All manuscripts should comply with the ICMJE guidelines for publication of clinical research and a completed CONSORT checklist must be included with all submissions.

Clinical trial registration	N/A
Study protocol	IRB protocol STUDY19050099
Data collection	Details included in manusript
Outcomes	N/A

Plants

Seed stocks	*Report on the source of all seed stocks or other plant material used. If applicable, state the seed stock centre and catalogue number. If plant specimens were collected from the field, describe the collection location, date and sampling procedures.*
Novel plant genotypes	*Describe the methods by which all novel plant genotypes were produced. This includes those generated by transgenic approaches, gene editing, chemical/radiation-based mutagenesis and hybridization. For transgenic lines, describe the transformation method, the number of independent lines analyzed and the generation upon which experiments were performed. For gene-edited lines, describe the editor used, the endogenous sequence targeted for editing, the targeting guide RNA sequence (if applicable) and how the editor was applied.*
Authentication	*Describe any authentication procedures for each seed stock used or novel genotype generated. Describe anyexp eriments used to assess the effect of a mutation and, where applicable, how potential secondary effects (e.g. second site T-DNA insertions, mosiacism, off-target gene editing) were examined.*

Flow Cytometry

Plots

Confirm that:

☒ The axis labels state the marker and fluorochrome used (e.g. CD4-FITC).

☒ The axis scales are clearly visible. Include numbers along axes only for bottom left plot of group (a 'group' is an analysis of identical markers).

☒ All plots are contour plots with outliers or pseudocolor plots.

☒ A numerical value for number of cells or percentage (with statistics) is provided.

Methodology

Sample preparation	Details included in manusript
Instrument	Details included in manusript
Software	Details included in manusript
Cell population abundance	Details included in manusript

Gating strategy | Details included in manusript

☒ Tick this box to confirm that a figure exemplifying the gating strategy is provided in the Supplementary Information.
